# Georgia Medical College Agreement

**Published:** 1891-08

**Authors:** Edw. Geddings, Wm. Perrin Nicolson, W. S. Kendrick

**Affiliations:** Dean Med. Dept. Univ. of Georgia; Dean Southern Medical College; Proctor Atlanta Medical College


### ARTICLES OF AGREEMENT BETWEEN THE MEDICAL COLLEGES OF THE STATE OF GEORGIA, 1891.

For the purpose of securing uniformity of action, and maintaining the rates of tuition, the Medical Schools of the State of Georgia, by their official representatives, hereby agree to the following rules for the government of the several institutions, to remain in force until formally annulled.

*First*—FEES—The regular fees for each course of lectures shall be as follows: First term—Matriculation Fee, $5.00; Lecture Fees, $100.00; Dissecting Fees, $10.00; total, $115.00. Second term—Lecture Fees, $100.00; Diploma Fee, $30.00; no Matriculation Fee, and no Dissecting Fee, unless the student dissects. Third term, when taken, will be given without any charge to such students as have paid for two full courses of lectures. This rate of tuition, which is a return to the fees of former times, will take effect with the beginning of the session of 1891-92.

*Second*—PAYMENTS—All fees, as far as possible, are payable at the opening of the term, though, it is left discretionary with executive officer of each institution, to make any business arrangements he may see fit for the payment of the balances during the term, provided always, that all indebtedness, including the diploma fee, shall be cancelled before the first of February.

*Third*—PAYMENTS BY NOTE—When circumstances shall arise that render the cancelling of the entire indebtedness of the student in cash impracticable, and when the faculty deem it expedient to accept the note of the party, the note taken must bear an endorsement that not only renders the same safe, but negotiable and collectible without trouble.

*Fourth*—BENEFICIARIES—There shall be no departure from the published rates of tuition except as follows: The Medical College of Georgia and the Atlanta Medical College, having at a former day received money from the State, are required by law to furnish free tuition to thirty students, the former twenty and the latter ten. These being governed by law and not subject to the control of the schools, it is agreed that the Southern Medical College be permitted to receive as many as ten students on similar terms, or at such reduction as they may see fit. Beyond the above number allotted to each school there shall be no reduction of any character whatever from the published rates of tuition, and it is further agreed that each college shall keep a record of the names of the students for whom such reductions are made, with the amount of the reduction and the reason therefor.

*Fifth*—Limit of Admission—The time of entrance of students must be so regulated that no one shall lose more than one-fourth of the regular session for any cause. This time of attendance is prescribed by statute in the State, and a penalty attached to its violation. It is further agreed that each school shall at intervals of one week call the roll of the class by its executive officer, and note absentees, and the amount of time lost by them ; and such roll call shall not be made upon stated days, but at the discretion of the Dean of the school.

*Sixth*—It is agreed that, for the mutual protection of the colleges against the charge of bad faith, and for the better understanding of business, the books of each shall be open to the inspection of the others as may be deemed advisable, and at the end of each term there shall be exchanged reports, showing the number of students in attendance, the amount of money received in cash, and the amount and kind of obligations taken in lieu of cash, such reports being made, if required, under the oath of the Dean of the school.

*Seventh*—Any school found guilty of a violation of this agreement shall be publicly noted as having failed in its obligation.

Edw. Geddings, M. D.,
Dean Med. Dept. Univ. of Georgia.

Wm. Perrin Nicolson, M. D.,
Dean Southern Medical College.

W. S. Kendrick, M. D.,
Proctor Atlanta Medical College.

